# Myorelaxant Effect of Bee Venom Topical Skin Application in Patients with RDC/TMD Ia and RDC/TMD Ib: A Randomized, Double Blinded Study

**DOI:** 10.1155/2014/296053

**Published:** 2014-06-24

**Authors:** Aleksandra Nitecka-Buchta, Piotr Buchta, Elżbieta Tabeńska-Bosakowska, Karolina Walczyńska-Dragoń, Stefan Baron

**Affiliations:** Department of Orthodontics and Temporomandibular Joint Dysfunction, Medical University of Silesia in Katowice, plac Traugutta 2, 41-800 Zabrze, Poland

## Abstract

The aim of the study was the evaluation of myorelaxant action of bee venom (BV) ointment compared to placebo. Parallel group, randomized double blinded trial was performed. Experimental group patients were applying BV for 14 days, locally over masseter muscles, during 3-minute massage. Placebo group patients used vaseline for massage. Muscle tension was measured twice (TON1 and TON2) in rest muscle tonus (RMT) and maximal muscle contraction (MMC) on both sides, right and left, with Easy Train Myo EMG (Schwa-medico, Version 3.1). Reduction of muscle tonus was statistically relevant in BV group and irrelevant in placebo group. VAS scale reduction was statistically relevant in both groups: BV and placebo. Physiotherapy is an effective method for myofascial pain treatment, but 0,0005% BV ointment gets better relief in muscle tension reduction and analgesic effect. This trial is registered with Clinicaltrials.gov NCT02101632.

## 1. Introduction

Apitherapy is the use of bee products, such as honey, pollen, propolis, royal jelly, bee venom, wax, and apilarnil (atomized drone larva) to prevent or treat illness and promote healing [1]. The roots of apitherapy can be traced back more than 6000 years to medicine in ancient Egypt. The Greeks and Romans also used bee products for medical purposes. This is described by Hippocrates (460–370 BC), Aristotle (384–332 BC), and Galen (130–200 AD) [2, 3].

Bee venom called apitoxin is a mixture of proteins: melittin (main component 52%), apamin, adolapin, phospholipase A2, hyaluronidase, histamine, dopamine, and protease inhibitor. It is a bitter, colorless liquid of density 1,1313 g/cm^3^ and pH 5,0–5,5 [4]. It has been used for at least 22 centuries, especially in Eastern Asia [3]. A honey bee (*Apis mellifera* L.) can inject 0,012–0.1 mg of venom via its stinger. The main component is melittin comprising 52% of venom peptides [4, 5]. Apamin is a mild neurotoxin that selectively blocks SK channels, a type of Ca^2+^-activated K^+^ channel expressed in the central nervous system. Dry bee venom consists of 2-3% of apamin [6]. Adolapin comprising 2–5% of the peptides acts as an anti-inflammatory and analgesic agent because it blocks cyclooxygenase. The most destructive component of apitoxin is phospholipase A2 (10–12%), because it degrades the phospholipids, which cellular membranes are made of. It also causes decreased blood pressure and inhibits blood coagulation. Hyaluronidase comprising 1–3% of peptides dilates the capillaries causing the spread of inflammation. Histamine (0,5–2%) is involved in the allergic response. Dopamine and noradrenaline (1-2%) increase pulse rate and protease inhibitors (2%) act as anti-inflammatory agents and stop bleeding [7, 8].

Bee venom therapy, also acupuncture (apipuncture), is used by some as a treatment for rheumatism and joint diseases, due to its anticoagulant and anti-inflammatory properties [6, 9–12]. BV also has an antiarthritic effect in patients with rheumatoid arthritis [4]. Anti-inflammatory and analgesic effect of BV may be explained by process of counterirritation, which increases pain thresholds sensitivity and reduces pain rating [3]. In Asia similar methods are used for pain reduction, for example, moxibustion (method of burning herbs to stimulate acupoints) [3]. Bee venom is also used to desensitize people allergic to insect stings and even for SAPHO syndrome treatment [13]. The treatment use of honey bee venom therapy is based on the long-known fact that bee keepers (who often get stung) very rarely develop arthritis or problems with their joints and muscles [1]. Bee venom therapy can also be delivered in the form of a balm, although this may be less potent than using live bee stings. Bee venom can be found in numerous beauty products. It is believed to increase blood flow therefore plumping the applied area, producing collagen.

The aim of the study was the evaluation of myorelaxant and analgesic effect of bee venom in patients with RDC/TMD Ia and RDC/TMD Ib.

## 2. Material and Methods

A parallel group, randomized double blinded trial was performed including 79 patients with painful RDC/TMD Ia and RDC/TMD Ib. Patients were allocated into one of two groups: 37 in experimental group and 42 in control group, by picking up an even or odd number from the envelope. Of these, 4 patients were excluded because of positive allergic reaction to bee venom substance (1 in experimental group and 3 in control group). Five patients did not attend control visits and two patients did not accept bee venom substance. The experimental group consisted of 34 subjects (males = 6 and females = 28) and the control group consisted of 34 subjects (males = 4 and females = 30) aged 22–34 years (average = 23 years).

The patients were examined using the RDC/TMD clinical and physical examination form and asked to fill in Oral Behavior Checklist and VAS scale. One person enrolled participants in the study and another dental practitioner assigned them to the interventions and control visits.

The main eligibility criteria for the participants were a temporomandibular disorder-positive RDC/TMD examination for groups RDC/TMD Ia and RDC/TMD Ib and patient agreement to participate in the experimental study. Exclusion criteria were bee venom allergy, hyperactivity to bee products, positive anamnesis of anaphylactic reaction after bee bites, skin wounds with skin surface discontinuation, RDC/TMD II, and RDC/TMD III.

Masseter muscle tension [*μ*V] at rest and in maximal contraction was measured in previously determined points. The points were marked on paper template and were repeatable.

**Figure 1:**
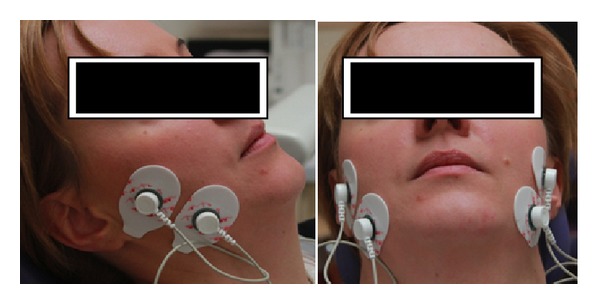
EMG Easy Train Myo (Schwa-medico, Version 3.1).

Bee venom is collected by electric stunning, without harming honey bees. To obtain pure BV it is necessary to remove impurities and lyophilize it. There are different methods of preparing pure BV, for example, capillary zone electrophoresis [14]. During one stung a honey bee injects one International Unit of Bee Venom (approximately 0,012 mg of liquid bee venom, after drying 10 *μ*g of pure bee venom). BV patients' group was given 0,0005% bee venom ointment for topical skin application in region of masseter muscle at the right and left side. Placebo group patients were given only ointment vehicle (Vaseline) in the same containers. Containers in both groups had even or odd number written on it (to allocate patient in one of the two groups). Patients were asked to perform allergic test first, by applying a small amount of the ointment (placebo or BV) on their forearm skin 24 h before therapy. In case of swelling, itching, or skin redness patients were asked to quit therapy. Each patient had been taught earlier how to perform simple physiotherapy with bee venom or placebo ointment. Patients were supposed to massage their masseter muscles 3 times a day during 2 weeks before control visit. Electromyography of both masseter muscles with Easy Train Myo EMG (Schwa-medico, Version 3.1) was performed (Figure 1). Rest muscle tonus and maximal muscle contraction values [*μ*V] were evaluated. Participants' skin was cleaned and rubbed with cotton ball moisture with alcohol. Six adhesive electrodes were applied symmetrically, directly over both masseter muscles insertions. Reference electrodes (two) were placed on the neck.

Data collected during electromyography were noted in Excel file. The results were analyzed with Statistica 7.0 for each group and Wilcoxon test analysis was performed (*P* < 0,05000).

## 3. Results

### 3.1. Changes in Muscle Tonus [*μ*V] and Muscle Pain Intensity in VAS Scale after Bee Venom Skin Topical Application

**Figure 2:**
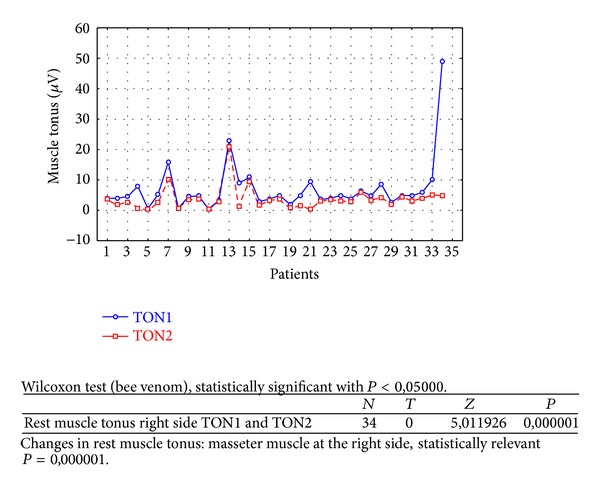
Changes in rest muscle tonus at the right side before (TON1) and after 2 weeks (TON2) with bee venom [*μ*V].

**Figure 3:**
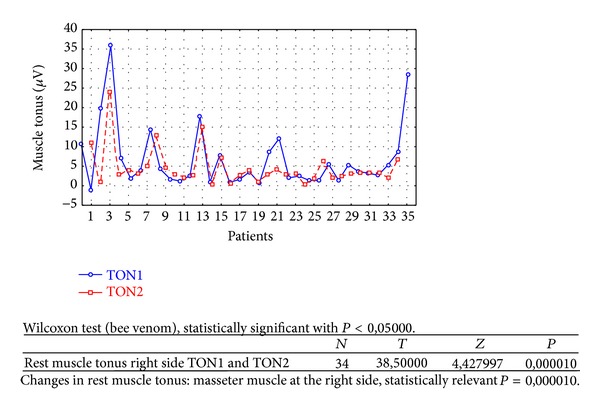
Changes in rest muscle tonus at the left side before (TON1) and after 2 weeks (TON2) with bee venom [*μ*V].

**Figure 4:**
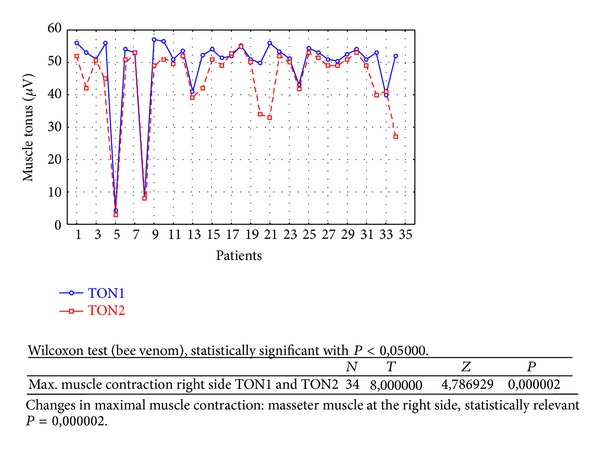
Changes in maximal muscle contraction at the right side before (TON1) and after 2 weeks (TON2) with bee venom [*μ*V].

**Figure 5:**
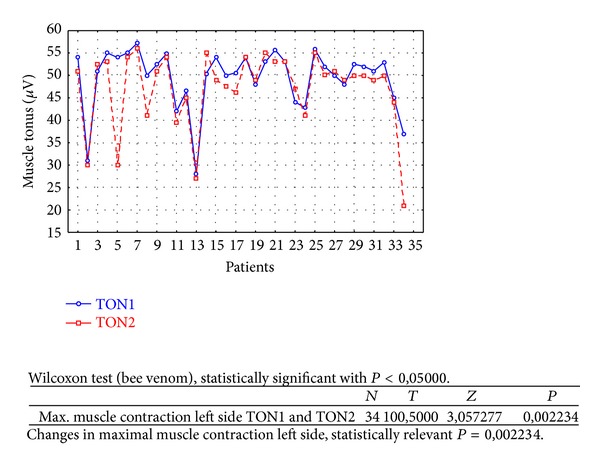
Changes in maximal muscle contraction at the left side before (TON1) and after 2 weeks (TON2) with bee venom [*μ*V].

**Figure 6:**
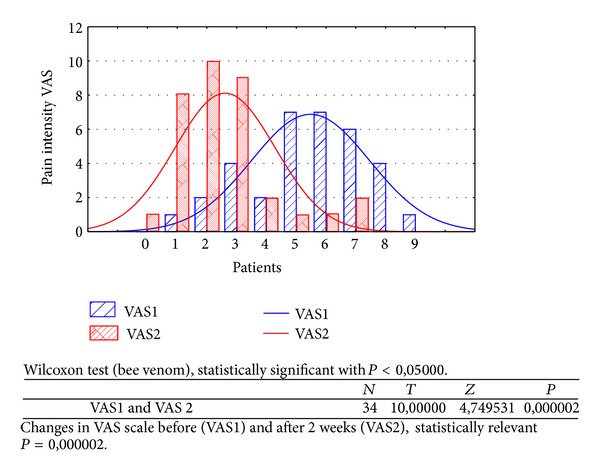
Changes in VAS scale before (VAS1) and after 2 weeks (VAS2) of bee venom.

1. Changes in rest muscle tonus [*μ*V] at the right side before and after 2 weeks of skin topical bee venom application are shown in Figure 2.
2. Changes in rest muscle tonus [*μ*V] at the left side before and after 2 weeks of skin topical bee venom application are shown in Figure 3.
3. Changes in maximal muscle contraction [*μ*V] at the right side before and after 2 weeks of skin topical bee venom application are shown in Figure 4.
4. Changes in maximal muscle contraction [*μ*V] at the left side before and after 2 weeks of skin topical bee venom application are shown in Figure 5.
5. Changes in VAS scale before (VAS1) and after 2 weeks (VAS2) of bee venom skin application are shown in Figure 6.

### 3.2. Changes in Muscle Tonus [*μ*V] and Muscle Pain Intensity in VAS Scale after Placebo Skin Topical Application

**Figure 7:**
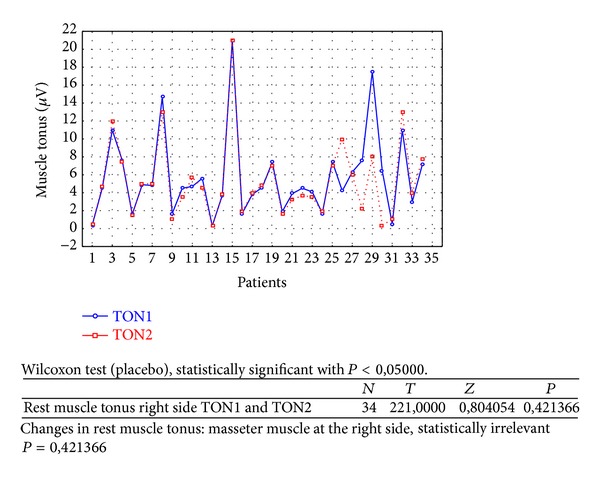
Changes in rest muscle tonus at the right side before (TON1) and after 2 weeks (TON2) with placebo [*μ*V].

**Figure 8:**
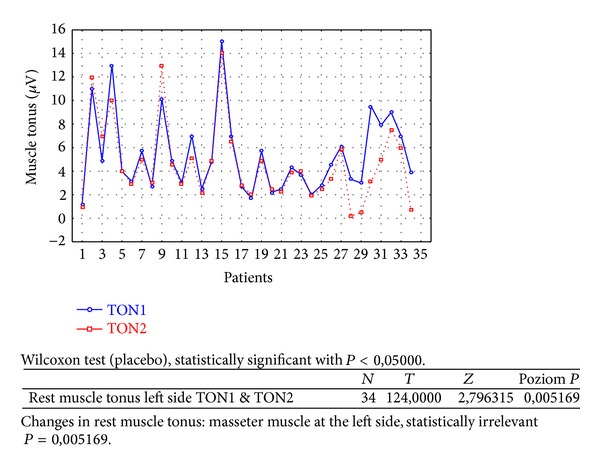
Changes in rest muscle tonus at the left side before (TON1) and after 2 weeks (TON2) with placebo [*μ*V].

**Figure 9:**
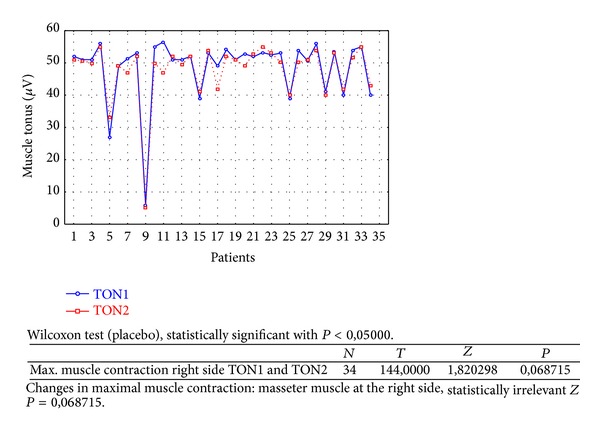
Changes in maximal muscle contraction at the right side before (TON1) and after 2 weeks (TON2) with placebo [*μ*V].

**Figure 10:**
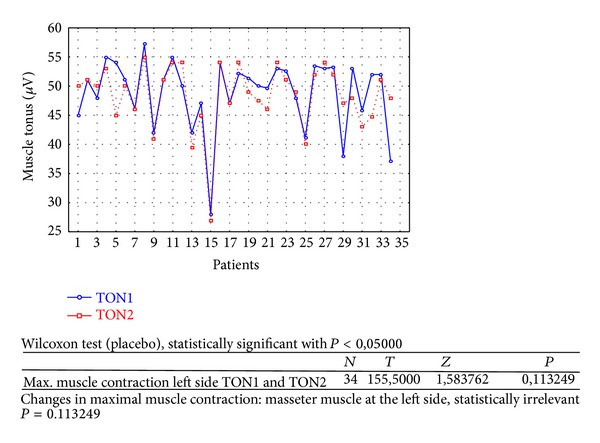
Changes in maximal muscle contraction at the left side before (TON1) and after 2 weeks (TON2) with placebo [*μ*V].

**Figure 11:**
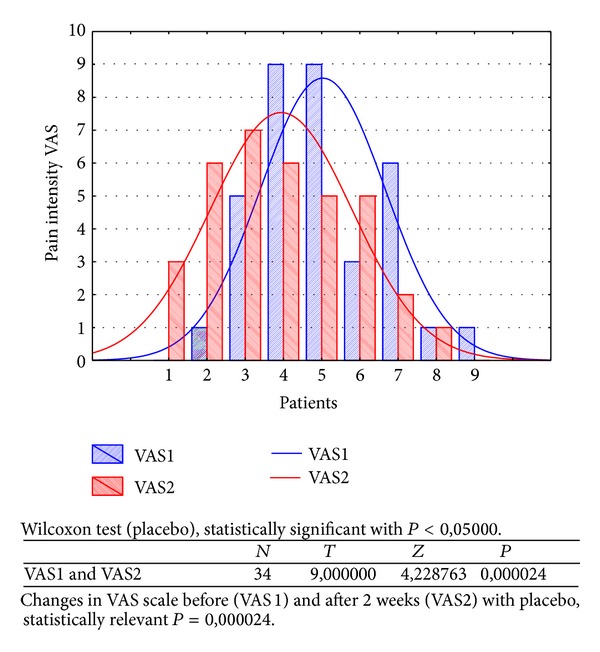
Changes in VAS scale before (VAS1) and after 2 weeks (VAS2) of placebo skin application.

1. Changes in rest muscle tonus [*μ*V] at the right side before and after 2 weeks of skin topical placebo application are shown in Figure 7.
2. Changes in rest muscle tonus [*μ*V] at the left side before and after 2 weeks of skin topical placebo application are shown in Figure 8.
3. Changes in maximal muscle contraction [*μ*V] at the right side before and after 2 weeks of skin topical placebo application are shown in Figure 9.
4. Changes in maximal muscle contraction [*μ*V] at the left side before and after 2 weeks of skin topical placebo application are shown in Figure 10.
5. Changes in muscle pain intensity in VAS scale before (VAS1) and after 2 weeks (VAS2) with placebo are shown in Figure 11.

#### 3.2.1. Rest Muscle Tonus Median Values [*μ*V]

Rest muscle tonus values before physiotherapy with topical application of bee venom (BV) are TON1BV right = 4,8 [*μ*V] and TON1BV left = 4,75 [*μ*V].

Rest muscle tonus values before physiotherapy with topical application of placebo are TON1 PLACEBO right = 4,55 [*μ*V] and TON1PLACEBO left = 4,45 [*μ*V].

Rest muscle tonus values after 2 weeks of physiotherapy with topical application of bee venom (BV) are TON2 BV right = 3,05 [*μ*V] and TON2 BV left = 3,1 [*μ*V].

Rest muscle tonus values after 2 weeks of physiotherapy with topical application of placebo are TON2 PLACEBO right = 4,3 [*μ*V] and TON2 PLACEBO left = 3,95 [*μ*V].

In both BV (Table 1) and placebo groups (Table 2) a decrease in muscle tension [*μ*V] was observed; it was statistically relevant for BV group, on both left and right sides (right *P* = 0,000001 and left *P* = 0,000010). For placebo group only on the left side, in rest muscle tonus, a statistically relevant decrease in muscle tonus was observed (right *P* = 0,421366 and left *P* = 0,005169).

#### 3.2.2. Maximal Muscle Contraction-Median Values [*μ*V]

Maximal muscle contraction values before physiotherapy with topical application of bee venom (BV) are TON1BV right = 52,4 [*μ*V] and TON1BV left = 51,5 [*μ*V].

Maximal muscle contraction values before physiotherapy with topical application of placebo are TON1 PLACEBO right = 51,95 [*μ*V] and TON1PLACEBO left = 51 [*μ*V].

Maximal muscle contraction values after 2 weeks of physiotherapy with topical application of bee venom (BV) are TON2 BV right = 49,25 [*μ*V] and TON2 BV left = 50 [*μ*V].

Maximal muscle contraction values after 2 weeks of physiotherapy with topical application of placebo are TON2 PLACEBO right = 50,4 [*μ*V] and TON2 PLACEBO left = 49,5 [*μ*V].

Reduction in maximal muscle contraction [*μ*V] was statistically relevant for BV group (Table 1) for both sides (right *P* = 0,000002 and left *P* = 0,002234). For placebo group (Table 2) maximal muscle contraction was reduced, but the change was not statistically relevant (right *P* = 0,068715 and left *P* = 0,113249).

#### 3.2.3. Muscle Pain Intensity Changes in VAS Scale

In both BV (Table 1) and placebo (Table 2) groups a decrease in muscle pain intensity (VAS scale) was observed (VAS1 = 6, VAS2 = 2); it was statistically relevant for BV group (*P* = 0,000002). For placebo group reduction in muscle pain intensity was also observed, but it was not so significant (VAS1 = 5 and VAS2 = 4), statistically relevant (*P* = 0,000024).

## 4. Discussion

Lee performed a review of the literature (11 RCTs) and confirmed the effectiveness of bee venom acupuncture, compared to acupuncture without any additional agents, in the treatment of musculoskeletal pain [4, 9, 10].

Efficacy of BV in myofascial pain therapy can depend on anti-inflammatory effect, which is a well-known fact for centuries [1, 6]. In our study patients evaluated their muscle pain intensity decrease in both groups; VAS BV decreased from VAS1 = 6 to VAS2 = 2 (Figure 6) and VAS placebo from VAS1 = 5 to VAS2 = 4 (Figure 11), which is probably the effect of local muscle irritation reduction and BV healing properties [4, 6, 11]. Intensive and long lasting muscle effort, when muscle expends more energy, evokes anaerobic exercise, probably in the mechanism of glycolysis (lowering pH and acidosis), which can be harmful for muscle cells and painful for a patient [8]. Research is needed to establish any relation between BV anti-inflammatory effects and glycolytic muscle irritation.

Day or nighttime bruxism produces great muscular damage and delayed onset muscle soreness one to two days after training, the reason of myalgia. Muscle effort in this case incorporates eccentric, concentric, or both muscular contractions; muscle soreness depends also on the way and direction of strength application [15]. All those changes can occur in patients with RDC/TMD Ia and RDC/TMD Ib during bruxism and muscle effort. Muscles undergoing heavy eccentric loading suffer greater damage when overloaded as compared to concentric loading [15].

Next possible mechanism of anti-inflammatory and analgesic effect of BV may be explained by process of counterirritation and pain threshold regulation, which increases pain thresholds sensitivity and reduces pain rating [3]. VAS changes in our study may be also connected with pain threshold sensitivity increase.

As a result of the study we have observed a reduction of muscle tension (rest and maximal contraction tonus) in both groups: BV and placebo. In BV group the reduction was statistically relevant on both sides, in rest muscle tonus (Figures 2 and 3) and maximal muscle contraction (Figures 4 and 5). In placebo group only the reduction of rest muscle tonus observed on the left side was statistically relevant (*P* = 0,005169) (Figure 8). Other parameters in placebo group were not statistically relevant (Figures 7, 9, and 10). On the basis of these data we can conclude that physiotherapy (masseter muscle massage) is an effective method of muscular tension reduction. Miernik et al. also have shown the physiological effect of different massage techniques applied in myofascial pain treatment [16]. Physiotherapeutic massage and muscle elongation induces blood perfusion and blood flow increase in muscle tissue [17–19]. It is a useful method for muscle tension and muscle pain reduction, but the efficacy of physiotherapy in our study was considerably increased by BV ointment (median BVTON reduction was 1,75; 1,65; 3,15; and 1,5 and median PLACEBOTON reduction was 0,25; 0,5; 1,91; and 1,5). Muscle tension can also be regulated by some BV proteins, for example, melittin. This peptide induces two-phase changes in rat papillary muscles: first it evokes an increase in the force of muscle contraction (15 min) and after that it inhibits muscle contraction for 30–45 min [20]. The effect is dose-dependent that is why further research is needed to evaluate influence of melittin on muscle cells contraction. That could be one of the possible mechanisms of bee venom healing properties on muscle tissues.

## 5. Conclusion

Physiotherapy is a very important and effective element of masticatory muscles myalgia treatment (RDC/TMD Ia and RDC/TMD Ib). Topical application of BV ointment provides excellent therapeutic effects: it reduces muscular tonus and muscle pain intensity and gives better relief to a patient than placebo (Vaseline). Local, topical BV application helps to minimize harmful side effects (as anaphylaxis) and teaches patients how to deal with muscle spasm, self-control, and biofeedback. Further research in that field is necessary to establish optimal dose (International Units of BV, %), a way of BV application, frequency, and duration of therapy.

## Figures and Tables

**Table 1:** Median values of muscular tonus before (TON1) and after 2 weeks (TON2) of topical skin application of bee venom.

Median values BV	Rest muscle tonus right side [*μ*V]	Rest muscle tonus left side [*μ*V]	Maximal muscle contraction right side [*μ*V]	Maximal muscle contraction left side [*μ*V]	VAS
TON1	4,8	4,75	52,4	51,5	VAS1 = 6

TON2	3,05	3,1	49,25	50	VAS2 = 2

**Table 2:** Median values of muscular tonus before (TON1) and after 2 weeks (TON2) of topical skin application of placebo.

Median values placebo	Rest muscle tonus right side [*μ*V]	Rest muscle tonus left side [*μ*V]	Maximal muscle contraction right side [*μ*V]	Maximal muscle contraction left side [*μ*V]	VAS
TON1	4,55	4,45	51,95	51	VAS1 = 5

TON2	4,3	3,95	50,4	49,5	VAS2 = 4
